# A Practical Treatise on Hæmorrhoids, Strictures, and Other Important Diseases of the Rectum and Anus, &c.

**Published:** 1825-04-01

**Authors:** 


### MEDICO-CHIRURGICAL

### REVIEW.

VOL. VI.] Analytical Series. [No. 20.

" Nec tibi quid liceat sed quid fecisse decebit
" Occurrat, mentemque domat respectus honesti."

VOL. II.] APRIL 1, 1825. [No. 4.

[NEW SERIES.]

### I.

*A Practical Treatise on Hæmorrhoids or Piles, Strictures, and other important Diseases of the Rectum and Anus: being, with some Additions, a Treatise, to which the Jacksonian Prize was adjudged by the Royal College of Surgeons.* By GEORGE CALVERT, Member of the Royal College of Surgeons of London, and of the Medico-Chirurgical Society, &c. Octavo, pp. 259, one plate. Sept. 1824. Callow & Wilson.

MR. CALVERT's work consists of seven chapters, *viz.* On Hæmorrhoids—Strictures of the Rectum—Morbid Contraction of the Anus—Prolapsus Ani—Fistula in Ano—Ulcers of the Rectum—and, finally, Excrescences about the Anus.

It is rather remarkable that, in this country, where monographs abound on almost every disease, there should be no regular treatise on the subject of hæmorrhoids—at least, none that has kept its place as a standard of reference. The most able and elaborate treatise which we know of, is that of Montegre—in the 20th volume of the *Dict. des Sciences Medicales*, and, also, in a separate and enlarged form. We strongly suspect that Mr. Calvert is not a little indebted to Montegre's work, though he makes little mention of that masterly performance. In this article, we shall draw from various sources, as occasion may require.

*Definition.* Various definitions have been given of this disease—most of them bearing reference to the etymology, and making the *sanguineous discharge* the prominent feature of the disease. Hippocrates, as every body knows, has taken this view of the case—"excretiones per ora venarum quæ sunt in ano hæmorrhoidas vocant." Mr. Calvert defines the complaint thus:—"a morbid state of the vessels of the rectum and anus, with pain, tension, &c. accompanied, or followed by the formation of tumours in those parts, and a flow of blood, frequently periodical." Montegre endeavours to separate the *accessory or accidental* symptoms from the *essential* feature of the disease, which he considers, (and we think justly so) as a sanguineous fluxionary determination to the vessels of the rectum and anus, bearing no small analogy to the menstrual afflux to the uterus in the other sex. "Il n'est qu'un seul symptôme de constant dans les hæmorrhoides, c'est une tension, une pesanteur plus ou moins douloureuse du siége et des parties environnantes, produites par la fluxion qui s'y est formée: *cette fluxion est l'affection essentielle,** et tout le reste ne doit être considéré que comme accessoire ou accidentel."

"In consequence," says Montegre, "of causes which it is often impossible to ascertain, there is established, at certain epochs, a sanguineous determination (fluxion sanguine) towards the extremity of the rectum; whence results, at first, simply a sense of tension and weight. This state, which has nothing painful in it, and which is often scarcely noticed, continues three or four days, and then subsides. Afterwards it is renewed, at longer or shorter intervals;—frequently, but not always, terminating in a discharge of blood of a bright red colour, spread over, but not mixed with, the fæces. This blood is generally produced by a kind of exhalation from the mucous membrane of the rectum, without any trace of erosion in the tissue. After frequent, and sometimes after a few repetitions of this fluxionary movement, tumours of greater or less volume, and more or less painful, begin to appear."†

Let us compare this with Mr. Calvert's description of the first approach of hæmorrhoids.

"The first attack of hæmorrhoids is generally very slight, and is not preceded by any marked constitutional derangement. There is some sensation of weight or fulness about the sacrum, and extremity of the rectum, extending, perhaps, to the perineum; and the sensibility of the bladder, urethra, &c. is sympathetically increased. This state continues for a short time, perhaps for two or three days, when in many cases a slight flow of blood takes place during the expulsion of the fæces, and smears their surface of a bright red colour. This flow never occurs in some individuals, particularly during the primary attacks; but when this

* "Hence it would appear, (says Mr. Calvert) that the immediate cause of hæmorrhoids consists in a preternatural determination of blood to the vessels of the rectum." P. 10.

† Montegre, p. 443, Dict. des Sciences Med.

is the case it forms, as it were, a crisis to the complaint, and the above-mentioned symptoms disappear at an earlier period.

"After a greater or less interval the same train of symptoms are generally renewed, but in a greater degree, acquiring strength by repetition. The sensations of weight, tension, &c. are more perceptible; some sympathetic phenomena are observed; the blood is discharged in greater quantity, and tumours of varied size begin to appear within or around the anus." 9.

The similarity of these two passages appears to us something more than accidental. In an eclectic treatise, however, Mr. Calvert is justified in drawing his materials from such sources as appear to him the best. He could not apply to a better store of facts, or to more accurate descriptions of phenomena, than are presented in the monograph of Montegre.

That hæmorrhoids are not a merely local affection, must be admitted by every one who has felt the complaint in his own person, or accurately observed it in others. The venerable Pinel enumerates among the general symptoms of an approaching hæmorrhoidal attack—"slight horripilation in the back and loins; occasional numbness of the lower extremities, hardness and contraction of the pulse, paleness of the countenance, dullness of expression in the eyes, dryness of the mouth, scantiness and high-colour of the urine, debility of stomach, flatulence, frequency of inclination to stool and urine, sense of bearing down about the anus and perineum, occasional mucous discharge from the rectum."—*Nosog. Philos. Vol.* II. *Cinquiéme Ed.* Who can doubt that these symptoms, so accurately pourtrayed, create a considerable "fluxionary movement" (as Montegre would express it) in the system, *preparatory* to the local demonstration, and not merely symptomatic of the local affection? Mr. Calvert, however, who gives nearly a copy of Pinel's symptomatology, attributes the *constitutional* phenomena to the influence of the topical portion of the complaint.

"In weak and irritable constitutions the influence of the local affection bears upon the aspect of the patient; the face is paler than usual, the eye appears sunk, from the dark circle beneath it; the abdomen becomes tumid, the feet swell, and, in addition to these appearances, there is a sensation of coldness with shivering, hard pulse, dryness of the mouth, &c." 9.

We grant, indeed, that when the local affection has continued a long time and is accompanied with much organic derangement, pain, or profuse discharge of blood, the constitution will evince symptoms of sympathy, such as are above described. But this is not the stage of things at which we are arrived, in this early part of the dissertation. In short, so far as we have yet described of the hæmorrhoidal movement in the system, it is to be regarded as a salutary, rather than a morbid affection. As we before observed, this movement only continues a few days and then subsides, leaving the person in better health than before.

*Hæmorrhage, Tumours, &c.* We have shewn that the discharge of blood is not essential to the hæmorrhoidal movement in the system; but the periodical return of this movement soon produces both that and several other phenomena, which are now to be considered. The hæmorrhage, in simple cases, is, as was before observed, by a kind of exhalation from the vessels of the rectum. Sometimes we see the blood spin out like a fine thread of red silk, when the patient strains at stool. Even this is not necessarily from a ruptured or patulous vessel, but may proceed from a dilated pore, as it were, of the mucous membrane. The jet stopped and the part wiped clean, and examined, even with glasses, no vessel can be seen. In more advanced states, however, and especially where the local affection is become complicated with permanent tumours, ulcerations, or other effects, the hæmorrhage is often from ruptured vessels. The quantity of blood discharged in hæmorrhoidal affections is often prodigious, and frequently produces great weakness for a time. This hæmorrhage assumes two forms—an active and a passive. The former, when moderate, is always salutary. It is characterised by some increased action in the pulse and temperature, and a relief of unpleasant sensations after the discharge. It does not, in general, take place but when the person is at stool, and does not accumulate, during the intervals, in the intestine. When the discharge is very large in quantity, it weakens the patient, and thus passes occasionally into the passive form, where it has proved not only dangerous, but sometimes fatal. One of the most frequent causes of these excessive sanguineous discharges from the hæmorrhoidal vessels, Montegre asserts to be, a common, but injudicious measure resorted to as a remedy—the application of leeches to the anus. *Passive* hæmorrhage from the hæmorrhoidal vessels may result from general or local causes. In the first case, it is characterised by debility, which debility is increased by the discharge—by a weak pulse—by languor of all the functions. It is not preceded by the tension, weight, and other symptoms which announce the active fluxionary movement—and it continues until proper measures are used to arrest it. The local causes of this passive discharge are, laxity of the vessels from repeated attacks of the active form—rupture of their coats—or organic derangements of the parts, as tumours, ulcers, &c.

It is not necessary, now-a-days, to discuss the nature of the fluid discharged. It is common blood from the *exhalents* of the part.

*Hæmorrhoidal Tumours.* These are considered by Montegre as forming no *essential* part of the disease, (if it deserve that name) but a mere consequence or effect of it. Passing over the vague and erroneous notions which were entertained by the ancients, and even till our own days, respecting the hæmorrhoidal tumours, we shall here only state what modern anatomists have ascertained.

"There are two kinds of hæmorrhoidal tumours, differing very materially, both in their appearance and structure. The first that I shall speak of are by much the most common, and constitute what are commonly termed piles. They are first seen in the form of small fleshy tubercles, generally of a brownish or pale red colour, and situate within the anus, or descending from the rectum. On examining them with the finger, they are found to have a somewhat solid and spongy feel, and, when cut into, present a surface more or less compact, and bloody, from which blood oozes, leaving the texture pale, and more relaxed.

"When these tumours are more external they are paler, and generally, also, more elastic and transparent; appearances which arise from the nature of the skin that covers them, and the serum with which their internal tissue is often infiltrated. These are sooner produced, and disappear more rapidly than the former.

"These tumours very often contain a central cavity, filled with fluid, or coagulated blood, which is of a brighter or darker red, according to the length of time it has been effused. The lining of the cyst is either smooth or granulated; and by the assistance of the microscope in the dead subject, after having forced into the arteries by which they are supplied with blood some fine and coloured injection, a few minute vessels may be traced, through which the fluid gradually exudes into the above-mentioned cavity, but there is evidently no connexion whatever with any of the larger vessels.

"This cavity is usually small, not exceeding the size of a pea, but it is sometimes large enough to contain several drachms of blood.

"More generally, however, there is no regular cyst, but the substance of the tumour is infiltrated with blood, which eventually becomes dark and coagulated. This blood does not appear to be the result of common extravasation, since it is not generally diffused, as in ecchymosis, but confined in separate patches of different shades, presenting a variegated aspect when the tumour is cut into; on closer examination it appears as if it were contained in dilated vessels, which traverse the tissue in the direction of the long axis of the rectum, so that, if the tumid part be divided longitudinally, they present numerous dark streaks through the substance, but, if the section be made transversely, small, roundish specks only appear.

"The manner in which the common hæmorrhoidal tumour forms is in general pretty uniform. The patient is first made sensible of its development by a peculiar pricking, stinging sensation, generally within or around the margin of the anus; and, on applying the finger to the part, it is felt slightly elevated, as if some newly-formed substance were forcing its way to the surface.

"The increase of these tumours, when once they have become in some degree permanent, does not take place in every direction; they elongate rather than expand, the body being usually of a conical form, and larger than the neck, which arises probably from their relative position with the surrounding parts. Sometimes more or less blood is exhaled from their surface; on other occasions a serous fluid only is exhaled, or they remain nearly dry; but in either case they generally disappear in a short time, and return again at an uncertain or regular period, increasing in size, and becoming firmer in texture with each repetition." 25.

This pathology agrees substantially with the investigations of the best foreign writers. Mr. Calvert quotes several modern and living writers of this country as still entertaining erroneous notions respecting the true nature of these tumours, some of which we shall notice presently.

It is well known that after repeated attacks of hæmorrhoids, especially if the tumours become strangulated by the pressure of the sphincters, they acquire a solidity and permanence that render them inconvenient, and sometimes very painful, leading to inflammation, ulceration, or, what is more common, a troublesome prolapsus ani.

"This permanent state of the tumours is owing partly to the development of the capillary vessels, gradually obliterating the interstices; and, in part, to the effused blood coagulating, and becoming organized; and hence the production of the condylomatous tumours, and the foundation of that irregular mass, which is found around the anus in those who have been long subject to hæmorrhoids, commonly termed the hæmorrhoidal excrescence, all of which are permanently solid, and can only be removed by the knife or the ligature." 27.

Such is the general character and such the structure of piles which, even to this day, are thought by many to be no more than dilated veins.

"Piles or hæmorrhoids," says Mr. Charles Bell, "are produced by a distention of the veins of the anus, and of the gut near the extremity. In a short time the distended veins are enveloped with a fleshy covering. The distention of the vessels causes a thickening of their coats, and the injury being still continued, there is a deposit of coagulable lymph around them; besides, there is sometimes an extravasation of blood which becomes organized. Thus the hæmorrhoidal tumours become a firm fleshy excrescence, with the extremity of the vein concealed within.

That vein, however, is burst, from time to time, and discharges blood; for when, by the continuance of the costive state of the bowels, the patient is forced to strain at stool, the blood is spurted out to some distance by the bursting of the extremity of the vein."

Sir Everard Home has viewed the structure of hæmorrhoidal tumours in the same light, and so did Dr. Baillie. Although more recent and more accurate investigations have proved these illustrious anatomists to be wrong, taking the thing in a general point of view, yet there are not wanting proofs that the pathology last detailed is *sometimes* correct, as the following passage from the venerable Pinel* will shew. "Lors de l'inspection cadaverique d'une femme anciennement hæmorrhoidaire, je remarquai quelques tumeurs vers l'anus, et des bosselures d'un rouge foncé dans la membrane muqueuse. On enleva avec soin cette membrane, et on trouva au dessous des tumeurs remplies d'un sang caillé. L'interieur de ces petites tumeurs se continuait dans de portions des vaisseaux qui avaient leur calibre ordinaire, ce qu'on reconnaissait en introduisant un stylat. Ces vaisseaux qui avaient toute l'apparence des veines presentaient alternativement un etat de dilatation et de leur calibre habituel. La direction de ces vaisseaux se continuait dans tous les sens, ce qui formait un vrais lacis vasculaire. Il me parait donc que ces tumeurs hæmorrhoidales n' etaient que des assemblages de varices ou des dilatations partielles de differentes portions." *Nos. Philos. tom* ii.

Mr. Calvert remarks that there is still one circumstance that has been overlooked, namely, the different appearance of these tumours in the living subject and in the corpse—a difference which he thinks has given rise to hasty and false conclusions.

"In examinations after death we almost invariably find, that where tumours exist within the anus or extremity of the rectum, they are found either to contain distended veins, or a condensed cellular tissue, but have rarely, unless where death has been produced suddenly, that humid, spongy substance, which is often observed during life, and which proceeds from the infiltration of fluids and the distended state of the capillary vessels. The cause of this is, I think, sufficiently obvious. Granting that, in the generality of such cases, there is an increased action, and greater development in the capillary vessels of the part, it follows that, in most cases, the degree of swelling must be almost wholly, or in part, owing to the accession of blood and white fluids to the part. Now, as this accumulation of fluids is the consequence of vitality, it must necessarily diminish with, or be altogether reduced by long pro-

* This case is erroneously attributed to Richerand by Mr. Calvert, at page 43.

tracted disease and death; and this is precisely the case with regard to the tumours that have already been described.

"In the dead subject then, these tumours which, during the hæmorrhoidal paroxysm, were fully distended, are found more or less collapsed, and the veins, which, prior to death, constituted but a small part of the swelling, and ramified chiefly between the body and envelopment, now occupy the centre, and are, both from their colour and size, the first objects that arrest the attention, when the latter part is removed for the purpose of investigating their nature. Hence it is that, even in cases where the above-mentioned swellings were formed during life by a congestion in the capillary vessels, the veins on dissection are often found enlarged, forming, together with the fine skin of the anus, or the villous coat of the rectum, the chief parts of the tumour." 36.

In other cases, where these tumours have been of long standing, a new substance is formed—the intestines are filled up from repeated accessions of inflammation, by which the parietes of the minute vessels are strengthened and elongated, and new matter deposited. Under these circumstances, the volume of tumours not being very materially increased by the accession of circulating fluids, continues nearly stationary after the phenomena of vitality have ceased.

*Hæmorrhoidal Inflammation.* This is no rare occurrence. The hæmorrhoidal tumours, when once formed, generally go on increasing with each paroxysm, and at length, by their size, cause great inconvenience in going up and coming down past the sphincter, when the patient is at stool. In this state they may become inflamed, and are then attended with exquisite pain of the throbbing kind, and accompanied by darting sensations at intervals, as if they were pricked with a lancet. "These symptoms," Mr. Calvert truly observes, "are much aggravated during the expulsion of the fæces, when the patient's sufferings often beggar description." There is more or less of symptomatic fever, and the whole system is exceedingly irritable. The inflammation may run so high as to end in suppuration or sloughing, with great sympathetic irritation of the bladder. Mr. Calvert illustrates this part of the subject by the following case.

"Mrs. ——, aged 22, after having for some time great inconvenience from the presence of some hæmorrhoidal tumours within the rectum, was attacked at the usual period of their enlargement with acute throbbing pain in the rectum, quick bounding pulse, retention of urine, and other symptoms of local and general irritation. A physician being called in, leeches to the anus, purgatives, &c. were prescribed, but the symptoms still continued to increase. At this time I saw her; her countenance was suffused, and distorted from pain, and although a woman of strong mind and great fortitude in general, she could not, in the present instance, avoid expressing her sufferings by loud and continued shrieks, particularly when the bowels were moved. The slightest motion of the body was productive of the most exquisite torture; and such was the sensibility of the inflamed parts, that an attempt to arrange the bed-clothes, or a light tread over the floor, added considerably to her sufferings.

"From the account of the nurse it appeared, that two dark-looking tumours had appeared at the anus, but at this time they were not visible; and the extreme sensibility of the parts rendered it quite impossible to make any satisfactory examination. A large quantity of blood was taken from the arm, and at the end of two days the violence of the pain had so far abated as to allow me to ascertain the state of the rectum. On carefully introducing my finger I could distinctly feel some loose soft bodies, partly detached from the mucous surface, and a few days after these were expelled without the slightest hæmorrhage. Three of these bodies, both in form and colour, very much resembled the common leech when partly distended, and appeared to consist of coagulated blood, inclosed by a dark membrane, which was too much disorganized to admit of any opinion being formed respecting its nature; another was of a rounder form, and appeared to have contained pus." 50.

*Leucorrhœal Discharge.* When mucous membranes are in a state of inflammation, or even irritation, it is their nature to pour out, first a thin acrid discharge, and after some time a mucous glazy secretion. This takes place very generally when hæmorrhoids have arrived at a certain height, and is much complained of, as soiling the linen, and keeping the patient uncomfortable. This is considered by Montegre as a veritable catarrh of the rectum.

*Fissures.* It not unfrequently happens, that when the anus is much obstructed by hæmorrhoidal tumours, a rupture of a portion of membrane takes place from the passage of indurated fæces, and the daily motion of the parts, together with the contact of excrementitial substances, prevent its healing. In consequence of this state of things, an ulcer is formed of a painful irritable nature, which gives the patient great suffering, especially after each motion. This naturally brings us to the subject of—

*Hæmorrhoidal Pains.* These are of different kinds, and are very minutely investigated and distinguished by Montegre. He classes them under four heads, viz. 1st, Those resulting from active inflammation.—2d, Nervous pains.—3d, Pains from fissures or ulceration—and 4th, Pain from chronic inflammation, or catarrh of the mucous membrane of the rectum.

1. The pains from active inflammation have already been described, when speaking of inflammation itself. They are distinguished by the accompaniments of heat, throbbing, tension, &c. and vary in the degree of intensity, from the most trifling to the most horrible sensations, ending even in gangrene.

2. *Nervous Pain.* This, Montegre observes, has not been noticed by any writers on hæmorrhoids, although it is one of the most afflicting accompaniments of the disease. Our author first described it in the GAZETTE DE SANTE, ten or twelve years ago, since which, M. Boyer has noticed it, and proposed the entire division of the sphincter ani as the remedy. The latter viewed it as depending on a fissure (Crevasse) in the rectum, producing that painful affection, spasmodic constriction of the anus. Montegre, who has seen the complaint frequently where no fissure or constriction existed, regards it as a symptom which may supervene on several affections of the rectum, but especially on hæmorrhoids. It takes place without any evident strangulation or inflammation, and, what is curious, it is relieved by *pressure.* It is characterised by intermissions, and by the rapidity with which augmentations or diminutions of the pain take place. Like the generality of nervous affections, it leaves the patient in the most extreme state of inquietude and depression. These pains frequently succeed inflammatory affections of the parts, and often remain when the cause that produced them is removed. They will continue for months or years—they will even become permanent, and embitter the remainder of life!

3. *Pain from Fissures.* It is to be borne in mind that fissures or rhagades will occasionally exist unaccompanied by pain. More frequently, however, they bring with them this disagreeable accompaniment. They will be recognized by the following symptoms.

"The patient experiences, on going to stool, a slight pain, the seat of which is always the same. This pain is, at first, so slight as to be scarcely perceptible. But after an interval, varying from a few minutes to an hour or more, it assumes an intensity that is compared by the patient to what might be expected from the introduction of a red hot iron into the anus. This anguish persists generally till the patient falls exhausted into a sound and prolonged sleep, from which he awakes free from pain. The state of tranquillity continues till he next has a stool, when he is destined to go again through the same train of sufferings. When this complaint has continued for a considerable time, the patient becomes affected with an habitual despondency, which is strongly depicted in his countenance. He emaciates rapidly—often avoids eating from the fear of having a motion. These fissures are sometimes cured spontaneously by the accession of acute inflammation ending in suppuration of the hæmorrhoidal tumour, at the base of which was situated the fissure. Sometimes also nature cures the disease by depriving the parts of their morbid sensibility, in a manner and by means unknown to us at present."—*Montegre.**

4. *Pain from Chronic Inflammation.* This differs but little from the species of suffering just described, except that the pain cannot be referred to a single point. It is more diffused, and is generally, but not always, accompanied by a mucous discharge or leucorrhœa from the parts. Like the distressing sensations from fissure, it succeeds each evacuation of the bowels, and is only relieved by sleep, which it too often prevents during the whole night. In that case the patient is doomed to suffer without interruption till the succeeding night!

*Contraction of the Anus.* This is an accident consecutive of hæmorrhoids, and may depend on three different causes.

1st. The accumulation and re-union, says Montegre, of tubercles around the lower part of the gut, and which protrude when the patient goes to stool, are very capable of producing a diminution of the passage, and a difficulty of voiding the fæces, especially when they are hard. Fissures are very liable to form under these circumstances.

2ndly. A progressive induration of the cellular tissue around

* A medical gentleman of this metropolis suffered, nearly two years from this complaint, and experienced exactly the symptoms described above by Montegre. The fissure, or rather a small chronic ulcer with hard raised edges, and a smooth surface, was discovered between two hæmorrhoidal tumours that always came down on going to stool, by an eminent surgeon, who removed the ulcer by excision. The operation was excessively painful, and the removal of the hæmorrhoidal tumours themselves was not deemed advisable at that time. A severe inflammation followed—the hæmorrhoidal tumours protruded—became strangulated—and sloughed. The sufferings of the patient, during the strangulation and sloughing of the piles, which occupied five or six days, were indescribable. A large raw surface was left, and two fistulous sinuses were formed in the gut, which required two successive operations afterwards. These all ultimately healed in the course of two months, and the patient has fortunately got completely cured of an hæmorrhoidal affection that had harrassed him for many years, and which for the last two years, had rendered his life a burthen to himself. During those two years he never passed a motion of calibre larger than a finger without exquisite torture; but since the operations, the gut has become nearly as free as at any period of his life. The complaint had produced much irritability of the bladder, which troublesome symptom has also disappeared with its cause.—*Reviewer.*

the extremity of the rectum occasions a gradual decrease of calibre in the gut, of an indolent nature, and which is borne by the patient, till the difficulty of voiding the fæces arrives at a certain height, when application for relief is made.

3rdly. *Spasmodic Constriction of the Anus.* This orifice, like most other natural passages in the body, is liable to spasmodic constriction, which here becomes a formidable and painful affection, from the nature of the functions of the part. It is to M. Dupuytren (observes Montegre) that we are indebted for the light which has been thrown on this complaint, of which he has seen many examples. The patients, in every instance, were what has been called of highly nervous temperament. As to the local affection, the sphincter ani is contracted with such force, that the fæces, when inclining to solid, are forced into the shape of small cylinders, and passed with intense pain. The same pain is experienced when the finger is introduced, with the view of ascertaining the nature of the disease. In several cases which M. Dupuytren examined with the greatest care, there was neither fissure nor inflammation to account for the pain. The eminent surgeon in question does not consider this curious affection as necessarily connected with a hæmorrhoidal disposition. He has found it in other natural openings of the body. Montegre, on the other hand, has sometimes seen it follow or accompany piles.

4thly. The fourth and last source of hæmorrhoidal pain is connected with *ulcerations, abscesses, fistulæ,* &c. of which it is unnecessary to say any thing in this place.

*Hæmorrhoidal Tenesmus and Prolapsus Recti.* These are common and well-known consequences of the disease under consideration. The afflux of blood to the parts causes a frequent nisus, or false desire to stool, and these repeated efforts produce, in the end, a prolapsus of the inner membrane of the gut. This protrusion, according to M. Chaussier's observations, is often formed by a kind of invagination of a portion of the bowel, a circumstance that merits attention, when the operation of removing the parts is contemplated.

Induration of the cellular tissue, and even scirrhus and cancer are, Montegre thinks, too often the melancholy consequences of repeated attacks of hæmorrhoidal inflammation.

Before proceeding to the etiology of the disease under consideration, we shall just enumerate the species into which Montegre divides hæmorrhoidal affections, viz, 1st, Hæmorrh. cœcæ, or blind piles—2ndly, H. fluentes, or sanguinolentæ—3dly, Hæm. tumentes,—4thly, Hæmorr. dolentes—5thly, Hæ-

cum contractione ani—6thly, Hæmorrh. cum procidentiâ ani—7thly, Hæmorrh. cum irritatione vesicæ urinariæ. This classification, is, we think, very convenient for teaching and acquiring a proper knowledge of the subject. We have already sketched out the nature and symptomatology of these different species, and shall now enter on an important consideration—the causes of hæmorrhoids.

*Etiology.* The causes are divided, by Montegre, into two classes—the antecedent or predisposing—and the occasional or determining. The *predisposing* are, physical constitution, generally transmitted hereditarily—climate—age—sex—habits and modes of life. Among the *occasional* or determining causes, Montegre reckons, season and temperature—food—constipation—sedentary and particularly literary habits—sombre affections of the mind—certain diseases—pregnancy—too light clothing of the abdomen—abuse of purgative medicines, especially of the aloetic kind—irritating lavements—venereal orgasms—external irritations, &c.

The experienced observer will acknowledge the correctness of this category—and the young practitioner should study the items seriatim. Mr. Calvert's Etiological Chart is nearly the same as Montegre's, but not quite so precise.

"A great variety of causes generally concur to produce hæmorrhoids; some of these are evidently pre-disposing, others have a more immediate and direct operation.

"Amongst the former, may be classed the peculiar situation of the hæmorrhoidal veins; hereditary disposition; a disordered state of the digestive organs, age, sex, and climate.

"The principal exciting causes of hæmorrhoids are, a torpid state of the bowels, fasting, sedentary habits, violent passions of the mind, irritation from worms in the rectum, from drastic medicines, excessive venery, &c." 52.

On the mode in which hereditary dispositions are transmitted from parent to child, too much has been written—especially as we know nothing at all about the matter, except the fact of such hereditary transmissions existing constantly before our eyes. We greatly fear that even Sir Everard Home's magnificent discovery of nerves in the funis and placenta of a seal, will hardly elucidate this mystery. We see features of the body, and capacities of the mind, unequivocally descending from parent to progeny—and why should not peculiarities of internal organization, with all their dispositions to morbid actions, also descend? We see no reason to the contrary—but examples of the fact are daily presenting themselves to the eye of the medical observer.

Of the natural constitutions of man, the bilio-sanguine is the most disposed towards hæmorrhoids, according to Montegre. Of the influence of climate in disposing to piles, we have little satisfactory evidence. The complaint is more common in this country, than in France, but this may be more owing to modes of diet, and passions of the mind, than to climatorial agency*.

The influence of age is more satisfactorily accounted for. Hæmorrhoids generally appertain to middle and advanced life—but then the bilious and melancholic temperament—the depressing passions—the maladies which dispose to piles; all these are the sad prerogative of the epoch in question. At this period, determinations of blood naturally take place towards the abdominal viscera, having run previously in currents towards the head and chest. In this respect, both sexes are alike concerned—but when the catamenial period has passed, females are necessarily and obviously more disposed to hæmorrhoids, than during the activity of the uterine system. It is a fact, however, that hæmorrhoids are by no means a rare occurrence in young people. We have seen them even in children. The writer of this article was subject to piles, at the early age of 19 years.

*Habits of Life.* These are, unquestionably, the most prolific of all sources of hæmorrhoidal affections. Inactive life and luxurious nourishment must bring on a train of diseases, or find some outlet for the superabundant fluids. Gout and hæmorrhoids are common consequences of this state of things. Hoffman, who practised forty years in Saxony, observes, that hæmorrhoidal affections had greatly increased in his time, from the progress of luxury and the increase of idleness and sedentary habits. A confirmation of this remark is found among people who have led an active life till a certain period of life, when, on leaving off business, and indulging in repose, they have become, for the first time, affected with piles.

*Food.* Alimentary substances of a stimulating quality have, in Montegre's mind, the property of disposing to hæmor-

---

* Mr. Calvert, who appears to have travelled in Greece and Turkey, has the following passage.

"The great frequency of hæmorrhoidal diseases amongst the Turks, may be traced to the indolent habit of sitting during almost the whole day, on warm soft cushions; to the peculiarity of their diet, which, in addition to their general habits, often produces an indolent and torpid state of the bowels; and perhaps, also, to an excessive indulgence in venery." p. 60.

rhoids—and that, by keeping up an afflux of blood to the digestive organs.

That the excitement of the stomach is *sympathetically* communicated to the colon and rectum is proved by the almost immediate effect of breakfast in producing an action in the bowels, in a state of good health. So the morbid excitement of the same organ by stimulating food and drink, produces a tendency to hæmorrhoids, through the same medium. This subject leads us to extract a passage or two from the work of Mr. Calvert.

"A disordered state of the digestive organs may predispose to the formation of different hæmorrhoidal affections, by producing that state of the constitution which seems to require the presence of a drain, if I may be allowed the expression, or a disordered action in some part of the body. The existence of this state, I have had frequent occasion to remark, and the opinions of Mr. Abernethy, where, in his valuable work on local diseases, he alludes to various anomalous affections produced and kept up by this cause, show how frequent it is. The following case illustrates this fact in a very striking manner:—

"A gentleman, of a delicate constitution, and who had impaired his health by irregular living, was affected with periodical attacks of pain in the head and face. He applied to a physician in the city, who prescribed something that gave him considerable relief. Soon after, he was attacked with pains in the abdomen, and diarrhœa, which continued to return, at intervals, for some months. At this time, an ulcer formed near the ancle from a slight scratch he had given himself, when asleep. In proportion as the ulcer enlarged, the preceding affections ceased; and, by adhering strictly to a prescribed diet, and varying the dressings, it at length healed. Pustules soon after appeared on different parts of the body, and these in turn gave place to a discharge of blood from the anus, with the usual symptoms of weight, tension, &c. Since then, he has been subject to hæmorrhoidal tumours, which appear to supercede the formation of other diseases, as, in other respects, he has continued to enjoy a tolerable state of health.

"The recurrence of diseased action, in cases similar to the preceding, would be attributed, by Mr. Abernethy, purely to a disordered state of the digestive organs; but, although this opinion may often be correct, it is more consistent with experience, and the known laws of the animal economy, to refer it to a general or constitutional affection. Independent, however, of the influence which a disordered state of the digestive functions may have upon the general health, and, through that medium, on the formation of hæmorrhoids, there are other circumstances connected with it, that tend, in a very particular manner, to the same effect. The constant passage of undigested and irritating aliment over the mucous surface of the large intestines, the frequent accumulations, and consequent purging that is produced, either naturally, or by the exhibition of medicines, act most powerfully where a predisposition already exists to the formation of hæmorrhoids." 57.

*Constipation.* Of the occasional or exciting causes of hæmorrhoids, M. Montegre properly considers constipation as in the first rank. It is not merely by the distention of the lower bowels, from the accumulation of hardened fæces, that irritation is produced there, and an afflux of blood determined to the part. The chemical qualities of these accumulations, Dr. M. thinks, tend also to increase the irritation, while their pressure on the meseraic veins produces congestion in the vessels of the rectum. Lastly, the efforts at expulsion gorge the vessels, and strain and protrude the parts in a manner eminently calculated to bring on the hæmorrhoidal paroxysm, where a disposition has previously existed, If constipation induces piles, it may be readily conceived how much it aggravates them when already formed. The parts are often lacerated by the straining, and then the serious and sometimes dreadful consequences of fissures, ulcerations, &c. ensue.

*Les Travaux du Cabinet.* By the phrase "*desk occupations,*" we can best render this into English. Montegre does not consider the *intellectual labours* of the cabinet as exciting causes of hæmorrhoids. They predispose, however, to this affection, by introducing into the system a morbid sensibility and nervous irritability which strongly conduce towards fluxionary movements or determinations of blood of every kind. Desk occupations operate in another manner. The sedentary habits, and sitting posture almost inseparable from them, produce constipation, and this soon excites piles, in the way before described.

*The Depressing Passions.* It is a curious fact that these will directly produce a fit of hæmorrhoids in those predisposed to them. On this, as on many other points of pathology and physiology, we are better acquainted with the *quo* than with the *quomodo*—in other words, we know the *facts*, but cannot *explain* them. Let us hear how Montegre explains the one just alluded to.

"The turbulent and depressing passions (la colère, la crainte, la tristesse habituelle) exercise a remarkable influence on the cœliac plexus, which governs the actions of the liver, gall-ducts and the whole of the vascular system therewith connected—including necessarily that of the lower bowels. The impression of these passions is felt at the epigastrium, by an uneasy weight and sense of constriction. This impression deranges the digestion, and often produces spasmodic vomitings, diarrhœa, and sometimes jaundice. The general effect of these profound emotions is to concentrate the circulation in the interior, and leave the cutaneous surface almost bloodless. In those who are disposed to apoplexy, this state often brings on such an event—and so it is with hæmorrhoids, in those predisposed to the complaint." 499.

The above explanation is, after all, little more than a faithful enumeration of phenomena. It is, however, the only explanation which can be offered in the present state of our knowledge.

*Abuse of Purgative Medicines.* Drastic purgatives, especially of aloes, colocynth, black helebore, &c. are well known provokers of piles. Montegre avers, that he has seen many instances of piles produced or aggravated by the habitual use of lavements of warm water, or endued with purgative qualities.

It was long an opinion among ancients and moderns that riding on horseback was prejudicial to hæmorrhoidarians. The fact is just the reverse. There is not a more certain preservative from piles than regular horse exercise. But if a person unaccustomed to equitation, takes this exercise in a sudden and violent manner—especially if he have the piles in the least protruded at the time—then, he may suffer from what, at another time, and more judiciously managed, would prove an excellent preventive. This fact is substantiated by our cavalry surgeons—and we need only refer to the testimony of Baron Larrey on this subject, for ample proof of the position now laid down.

The repeated application of leeches to the anus Montegre has seen bring on hæmorrhoids. This may be true, and yet leeches may be proper when inflammation supervenes on the common hæmorrhoidal movement.

It is no uncommon thing for those who are afflicted with painful, or permanently protruded piles, to furnish themselves with a chair or cushion, pierced with a hole in the middle, in order to prevent any pressure on the parts about the anus. This, though productive of present ease, is, according to Montegre, an injurious practice, as it favours the production and continuation of the hæmorrhoidal tumours. It stands to reason, indeed, that parts which have a tendency to protrusion should be supported rather than have all artificial support withdrawn. Indeed, it is often essentially beneficial, in these cases, to keep a pad on the anus, by means of a T bandage, and that braced pretty tightly, to prevent the descent of the hæmorrhoidal tumours.

*Diagnosis.* There is little difficulty in distinguishing piles from other affections that may bear some resemblance to them—but there is some difficulty in ascertaining whether they are of constitutional origin, or are only produced by temporary or accidental causes. The distinction must be drawn from an investigation of the symptoms, the mode of life, the age of the patient—and particularly from the causes that produce them.

*Suppression of the Hæmorrhoidal Discharge.* Cullen gave a very unguarded opinion respecting hæmorrhoids. He considered the disease as rarely of a constitutional, and generally of a local nature. He was consequently of opinion that the disease was, in all cases, to be removed as speedily as possible, and seemed to apprehend no danger from a suppression of the discharge. Now this may be all very well in sound constitutions; but where there is any defect in the system, or weak organ in the body, we should be careful how we suddenly stop the hæmorrhoidal movement and discharge. Montegre quotes, from various authorities, a great number of disorders brought on by the suppression of hæmorrhoids, as fevers, inflammations of internal organs, hæmorrhages, the neuroses, and organic diseases. Mr. Calvert gives an instance, in his own practice, of gastric fever, caused by the application of cold water to the anus during an attack of piles. Mr. Howship states, that a gentleman, who had a periodical discharge of blood from some hæmorrhoidal swellings, was induced by the advice of a quack, and contrary to the opinion of his surgeon, to apply a strong vitriolic wash. This cured the discharge, but the gentleman died in three days afterwards of gout in the stomach. Apoplexy has also been known to succeed similar acts of imprudence. The father of physic, Hippocrates himself, observed that the hæmorrhoids preserved the constitution from a great number of diseases. The oftener the hæmorrhoidal attack is renewed (as Mr. Calvert properly observes) the more liable it will be to recur, "and the greater will be the risk of endeavouring to effect a sudden cure." On this account it is highly desirable to ascertain the cause or causes of the complaint, and remove them. We now come to an important division of our subject—the Therapeutical.

*Treatment.* Montegre well observes that the treatment of the hæmorrhoidal movement in the system is a different thing from that of its local *effects* or *consequences*—as the discharge, the tumours, &c. We shall first speak of these accidents or consequences. And we agree with Montegre that, when a medical man is consulted respecting piles, he should invariably examine the parts carefully, since nothing is so fallacious as the descriptions given by the patients themselves, of which we have seen many instances.

A. *Treatment of the Sanguineous Discharge.* While the discharge from the hæmorrhoidal tumours is moderate—returns at intervals of some distance—and leaves no unpleasant effects, it should not be considered as a *disease,* but only a salutary adjustment of the constitution, attended, it is true, with inconvenience, but with more than counterbalancing advantages. From the moment, however, that the discharge becomes excessive, no time should be lost in restraining it. To judge of the excess, we must look to the *effects* rather than the *quantity* of the discharge. Some people will lose great quantities of blood daily in this way, and yet be in perfect health; but whenever the discharge is followed by pallor, debility, or spasms, it is then in excess, and should be restrained.

Like most other hæmorrhages it may appear in the *active* or in the *passive* form. In the active form, we should endeavour to divert the determination of blood from the hæmorrhoidal vessels, as well as lessen the local hæmorrhage. The body should be laid horizontal, and kept cool and quiet. These means alone will often succeed, without other remedies. If not, blood should be drawn from the arm—cooling drink should be ordered. If these means do not succeed—and if the patient become weak and nervous, the discharge still continuing, cupping-glasses, with or without scarificators, may be applied to the loins or hypochondriac regions. The ancients were in the habit of applying ligatures on the extremities, in cases of profuse hæmorrhage, with the view of retarding the blood in the veins. The moderns neglect this measure, though it is reasonable to suppose that it might be serviceable in conjunction with others.

Revulsives, as sinapisms, blisters, and other applications to the surface are proper, in order to draw the blood to the surface. These means failing, we must have recourse to local applications, as cold and astringent lotions, injections, &c. or the rectum may be plugged.*

* The ancients, and even some of the practitioners of the last century, applied the actual cautery, in these cases. Sultehes states that he saved the life of a patient by this means. The operation requires address—and is not practicable, unless the spot whence the blood issues can be brought into view.

In the passive forms of hæmorrhoidal hæmorrhage, we must exhibit the mineral acids with bark, steel, or other tonics. The superacetate of lead and opium, however, will be found the most powerful remedy in this as in most other hæmorrhages.

*The Hæmorrhoidal Tumours.* Of all the remedies for protruding piles, pressure is, unquestionably the best. In fact, no man need be troubled with external piles who, after each alvine evacuation, washes the anus with cold water, and applies pressure to the piles, by means of a T bandage—that is, one handkerchief tied round the loins, and another crossing between the thighs, and drawn tight, with a piece of soft sponge or a pad of linen pressed on the anus. We have known men, who were unable to crawl about their rooms, in consequence of painful protruding piles, quickly rendered capable of walking out without inconvenience; and the more they walked or rode, the better for the piles. When the hæmorrhoidal tumours, however, are internal, and only protrude on going to stool, the case is altered—and if the constitutional means which shall be hereafter pointed out, do not succeed in correcting the hæmorrhoidal tendency and diminishing the tumours, we think there should be no time lost in removing them by an operation, in order to prevent many painful or even dangerous consequences, which too often ensue, when they are suffered to remain and keep up constant irritation in the rectum.

The ligature and the knife are the two means now used almost exclusively for the removal of hæmorrhoidal tumours. Great differences of opinion have prevailed, and still prevail respecting the more eligible of these two plans. Mr. Calvert quotes the following passage from one of our latest writers, and one of our most active practitioners in this disease, of the present day—Mr. Copeland.

"'But I am sorry to say, (observes Mr. Copeland) that although I have repeatedly succeeded to the utmost of my wishes, in curing the disease, by the application of a ligature, this success has not been so uniform as to establish it in my mind, as an operation always to be recommended. In one instance the patient very narrowly escaped death; in another, very serious symptoms were produced, and in a third the operation nearly proved fatal. I have also heard of one or two instances where the life of the patient was destroyed by freely tying off the hæmorrhoidal excrescence.'" 99.

Now we know that Mr. Copeland's practice is, and long has been, almost invariably the *ligature*. Mr. Copeland's success, too, by the ligature, is entirely owing to his following the precept of Galen, laid down so many hundred years ago—namely to draw the thread as tight as possible, so as to completely destroy the vitality of the tumour. We think that it must have been inattention to this important point that has created so much prejudice against the ligature. Le Dran observes that, besides the extreme and almost insupportable pain caused by the ligature, it is possible that the inflammation extending along the rectum may reach the other intestines, and give rise to those effects which take place in hernia from strangulation of the bowel. "It is therefore better to cut them off in succession." Mr. Abernethy also prefers the knife.

"Yet it has appeared to me, that tying hæmorrhoidal excrescences is productive of all that temporary distress which is observable in what I have termed their natural cure; and as there is a general disorder in the functions of the alimentary canal in all such cases, the irritation occasioned by the ligature aggravates this habitual disorder, and produces sometimes very alarming symptoms."* 98.

"Montegre condemns the ligature for the following reasons—1mo. Because the operation is often very difficult, and always very painful—2ndo. Because the tumours sometimes resist the ligature, and instead of falling off, take on an ulcerative process—3tio. Because, as the tumours can only be tied in succession, the irritation produced by the first operation increases the swelling and inflammation of the remaining tumours—4to. The ligature may produce all the effects of strangulation of the gut, as in a case reported by Petit in his Chirurgical Works. For these reasons the ligature should be abandoned. It appears to us that all the above objections of Montegre are founded on the inefficient mode of applying the ligature, and that few or none of them are valid, provided the thread is drawn to a proper degree of tightness at the beginning. Let us hear what Mr. Howship has to say.

"'Many patients are not able to overcome the alarm they feel at the idea of cutting, none of which apply to the mere tying of a tumour. As relates to the surgeon, a stronger reason presents itself in the superior safety of the ligature, compared with the knife. The hæmorrhage, after excision, is sometimes very alarming, and has repeatedly proved fatal. In both modes of operating, the removal of the disease is effected with equal certainty, provided the operation is properly performed. It is supposed, also, that the ligature should be preferred, because it is followed by a greater degree of inflammation, producing an effusion of lymph, which is afterwards only partially absorbed; and that, in consequence, the parts previously relaxed become more firmly consolidated.'" 99.

* "Abernethy's Surgical Works."

Mr. Calvert does not agree with Mr. Howship, in thinking that the ligature is the safer method of the two—or that fatal hæmorrhage has frequently supervened upon the use of the knife. Indeed, Mr. C. appears to think that there are but two or three well authenticated cases of fatal bleeding under such circumstances. We could furnish, from our own memory, more than this number. Mr. Abernethy does not appear to have met with any serious hæmorrhage in his own practice.

"'It is now twenty years since I first began to remove them freely with the knife or scissors, and I have never met with any circumstances to deter me; whilst the relief of suffering, which the operation has afforded to some, and the scarcely to be expected and complete cure which it has effected in many, has been highly gratifying.' "* 105.

Montegre observes, (and Mr. Calvert has copied him†) that the danger of hæmorrhage in excision, arises from opening a varix in mistake for a hæmorrhoidal tumour. "It is of great consequence, says he, not to commit this error, which may be so fatal in its results." Zacutus Lusitanus relates the case of a young man who died of hæmorrhage after such an operation; and Denis states that the son of Charles the Fifth died in the same way. Gottlieb gives us the case of a man, 40 years of age, robust and sanguine, and always enjoying good health, who became affected with blind piles that protruded. The surgeon in attendance, one day opened one of the piles with a lancet; and as very little blood issued, he extirpated it with the knife. The gut immediately retracted, and the pain was excessive, especially when the alvine discharges took place. Blood was at first discharged, and afterwards pus. Tenesmus followed, with daily hæmorrhage, by which he was tormented during four years, without experiencing the least relief from medical or surgical treatment. Disgusted with doctors, he discharged them all, and trusted entirely to emollient glysters with opium. These produced only temporary relief, and the wretched sufferer dragged on a miserable existence till the age of 52, when he died, dropsical, having used more than two thousand glysters!

The modus operandi, both in excision and with the ligature, we shall give in Mr. Calvert's words, as the directions are very proper.

"The mode of operating for the removal of hæmorrhoidal tumours, must depend, in some degree, upon their position, size, and form. When they are nearly isolated, little or no difficulty can attend their removal: but when they are more extensively connected with the sur-

"* Surgical Works, Vol. ii." † See page 102.

rounding parts, or form an irregular mass around the margin of the anus, some previous consideration is required to effect their removal with nicety and advantage.

"Having placed the patient upon his knees and elbows, and previously cleared the rectum by injecting into it some warm water, let him strain, so as to force the tumours downwards, whilst an assistant holds aside the nates. When, by these means, the tumours are more exposed, the surgeon is to grasp that which he intends to remove first between his finger and thumb, and draw it gently forwards, or a hook, if requisite, may be employed for that purpose; and having exposed the whole of the tumour, it is then to be separated close to its base, with the probe-pointed bistoury, or the common scalpel. If there are more they may be removed in the same way, but this is not always necessary, as the smaller ones often disappear altogether when the others have been extirpated.

"If the tumours form an irregular and projecting mass around the anus, it is better to include the whole in two ligatures, tied at opposite points. The ends of the two ligatures should then be gently pulled, so as to draw the whole mass a little forwards, and the whole included by the ligatures separated with the bistoury or scalpel. The position of the patient will withdraw the surrounding skin, and in the latter case effectually prevent too much of it from being removed with the tumour.

"Sometimes also it is necessary to employ the scissors, but they should not, I think, be used unless when the tumours are very small, or it is inconvenient to use the knife, because, from the contusion which they undergo in this case, the wound is necessarily more disposed to suppurate. When the same operation is to be performed upon a tumour completely within the rectum, the wooden gorget, or director to guide the incision, and prevent the sides of the gut from being wounded. The improved speculum ani will, in this case, be found useful to ascertain the situation of the tumour, and enable the operator to place the director in the proper position. When this is done, the common bistoury must be introduced along the inside of the fore finger, carried over the neck of the tumour, and the division effected by drawing it forwards firmly along the director.

"In applying the ligature, the patient must be placed in the same position as in the former case, and, if possible, the whole of the tumour that is to be removed exposed in the manner already mentioned. If the tumour is large, or very broad at the base, a double ligature should be passed through it near to its basis by means of a curved needle; but under other circumstances a single ligature will answer every purpose. In either case, however, great care should be taken that the ligature is applied with sufficient force to prevent any partial communication between the parts it is intended to separate; and, as the degree of irritation must be, in some degree, proportionate to the quantum of substance compressed by the ligature, it is not advisable to operate upon several at the same time.

"Previous to performing either of these operations, it is necessary, on the one hand, that the bowels are sufficiently unloaded, and, on the other hand, that the medicines which have been given for this purpose have ceased to operate. After the operation is completed, an opiate should be given to allay irritation, and to diminish the peristaltic motion until the rectum is in a better state to receive and expel the contents of the bowels. This plan of treatment, however, is less necessary after the application of the ligature than when the knife has been employed, because, in the one case, our object is to produce a speedy cicatrization, whereas, in the other, suppuration is a necessary result; and, besides, the greater probability of danger from inflammation may require that the action of the bowels be not too much suppressed." 110.

A combination of the two methods has been proposed, and executed by some modern surgeons. Sir Everard Home, who had seen many bad effects from the ligature, recommends that the tumour be taken up with a double ligature, and the intermediate portion afterwards divided. In this mode of operating, he states that the subsequent symptoms are much less violent. Mr. Calvert makes a very just observation on this proposal.

"But it is very evident that it is founded upon a wrong principle, for if the ligature be tied sufficiently tight, all communication with the substance of the tumour is necessarily cut off, and no advantage whatever can therefore be gained." 112.

Mr. C. Bell recommends a nearly similar operation, viz. to tie the tumour at its base, and cut off the convexity with the scissors. The following are his directions.

"'The patient resting on his knees, the surgeon holds aside the nates. The surgeon, taking hold of the tumour betwixt his finger and thumb, draws it down, so as to expose the base of it. Now let him pass the hair-lip-pin across the base of the tumour, take off the steel point from the silver pin; over the pin, and consequently fully over the tumour he is now to draw his ligature; he is to draw as much as the patient can bear, without excessive pain: with one motion of the long curved scissors he is to remove the tumour which is thus included in the ligature.

"'The object of the first part of this operation is to restrain the bleeding, and to keep the membranes in contact, that they may adhere, and be consolidated. The advantage of the method is the ease with which it is done, and that the pin may be withdrawn on the first rising of the pain and tension. In the succeeding morning, or in the evening of the same day in which the operation is performed, the pin may be withdrawn if there come pain and tension on the part, for its purpose is answered. But this will not in general be necessary; the pin and the ligature may be permitted to remain until the parts go through the whole process of inflammation. The effect of this operation with the knife or scissors, thus performed, is the adhesion and consolidation of the loose membrane, and the obliteration of the vein which bleeds.'"* 113.

* Galen recommends a nearly similar operation.—*Rev.*

Mr. Calvert objects, of course, to Mr. Bell's proposal, considering the cutting of the tumour as quite unnecessary, provided the ligature be drawn sufficiently tight in the first instance. "If the ligature be properly applied, the vitality of the tumour is as much destroyed as if it were really separated." In conclusion, Mr. Calvert observes, that a contracted state of the rectum is not uncommon, after large tumours have been extirpated from these parts, if the case be left entirely to nature. This affection, however, is readily cured—and still more readily prevented, by the use of a bougie, introduced daily at first, and afterwards gradually left off.* P. 115.

*Treatment of Inflamed Piles.* Montegre, as we before observed, decidedly objects to the application of leeches to inflamed hæmorrhoids, and thinks that this measure tends to produce determination of the blood to the parts. In this, we cannot entirely agree with Montegre, though we know, from personal experience, that there is not so much advantage derived from leeching in this as in some other local inflammations. Our author justly remarks that there is always some degree of strangulation accompanying, or rather producing, the inflammation, and, therefore, attempts should be made to push up the tumours within the sphincter, if they be not too painful for that operation. Emollient cataplasms and fomentations are then proper. Injections, if they can easily be thrown up, are necessary to clear the bowels. When the inflammation is intense, cold lavements cannot be employed—but when the inflammation is somewhat subsided, there is no more powerful or efficacious remedy, in Montegre's experience, than injections of cold water. We shall speak more in detail of this remedy, when we come to the treatment of *painful hæmorrhoids.* That we should be very cautious in using any violence with inflamed piles, may be inferred from the melancholy case related by Zacutus. A man, being greatly annoyed with inward piles, which obstructed the rectum, and prevented his having a motion for several days, was visited by an officious and awkward person, who, in attempting to introduce the pipe of an injection syringe, lacerated one of the tumours. The immediate

* Here Mr. Calvert finishes the treatment of hæmorrhoids; and of him we must now take our leave. The subject, however, requires further prosecution; and we must take Montegre for our text during the remainder of this article. We may observe here, that Mr. Calvert's observations are, in general, very judicious, and that his Essay on Piles is the best in the English language. The other subjects in this volume, we shall notice in a succeeding number of our Journal.

consequence was excessive pain, then gangrene, and, in two days, death!—Gassendi records the death of Pieresi, the illustrious Mæcenas of his day, by a similar accident. Montegre properly observes that, in the case of inflamed piles, the patient should be allowed only diluent drinks, and such farinaceous support, as produce very little alvine discharges—these last being always very painful, under the best management. A medical friend of ours, who is subject to painful and inflamed piles, confines himself, on these occasions, to water gruel, for some days, taking an opiate every night, to prevent any evacuation from the bowels, till the tumefied and irritable state of the hæmorrhoids subsides. He then takes gentle aperients, or emollient injections. We do not vouch for the general utility of this practice, especially the opiate. Some surgeons are in the habit of making a very free incision, with a lancet, into the inflamed and protruded pile, with the view of discharging a quantity of congested or coagulated blood—and, they say, with considerable success. We cannot speak, from much experience, of this plan, but we have seen it done, without any bad consequences resulting.

*Fissures.* In the third volume of the Analytical Series of this Journal (Sept. 1822) we gave some account of M. Boyer's paper on spasmodic stricture, and violent pain of the rectum, produced by fissures or crevasses in the gut. Montegre differs, in some measure, from Boyer, and looks upon these fissures as only contributing causes of the excruciating torments which harrass some hæmorrhoidarians, and which he considers as essentially of a nervous character. He founds his dissent on the following circumstances, viz. 1st—he has seen these fissures exist, without producing any violent pain—and, 2ndly, he has seen cases of excruciating pain, where no fissures obtained. He, therefore, considers them as accidental, but aggravating concomitants on painful or nervous affections of the rectum.

In respect to the remedy which Boyer recommends—a complete division of the sphincter ani, M. Montegre avers that "this cruel operation is not always a certain remedy for the complaint. I have seen a patient who was twice operated on, and without any permanent relief." Montegre's remedy for this cruel disease will be seen presently. In the mean time, it is proper to apply surgical means to these fissures. They are sometimes produced, or kept up, by the tumours in their neighbourhood, and the removal of these last will cure the fissures. Sometimes they may be cured by caustic—by the cautery—or by actual excision. We have already mentioned a case of this kind, where the fissure had become a chronic circular ulcer, with raised and indurated edges.

*Treatment of Anal Leucorrhœa, or White Mucous Discharge from the Anus.* This is the "hæmorrhoides albæ, mucosæ" of nosologists. It is a frequent accompaniment of piles —but it is not necessarily so, and, consequently, is not to be confounded with hæmorrhoids. If inflammation be present, this must be reduced by the usual means, and the discharge afterwards cured by slightly astringent and detergent injections, with the internal use of balsam of copaiba, oil of turpentine, or cajeput oil.

*Treatment of Nervous Hæmorrhoidal Pains.* Our readers will remember the description which has been given a few pages back, of this terrible disease. They will remember that the pain is of an intermitting character—that is, it is renewed each time the patient goes to stool, and generally ceases when sleep takes place. Montegre was led to the discovery of the efficacy of cold injections in this disease, by observing that when patients affected with the complaint, happened to discharge their fæces while bathing in a river, or the sea, they sometimes escaped the torment which ensued, when they used a common commode. But, unfortunately, this does not always, nor even generally succeed, and it is often an impracticable measure. After giving a list of the various remedies which have been recommended for this formidable malady, including hyoscyamus, belladonna, solanum nigrum, stramonium, opium, the preparations of lead, spirit of wine, vinegar, balsam of sulphur, &c. all of which have been highly praised, though few of them deservedly so, our author comes to COLD WATER, which, in his experience, is far superior to them all. It is to be used either as a lotion, a "*douche ascendante*," or an injection. Montegre observes, that he could relate a great number of instances, where the success of this remedy was indisputable, but contents himself with the two following.

*Case* 1. "A man, 34 years of age, of good constitution, but hæmorrhoidinary from an early period, had, during the last few years, experienced long and painful attacks of the complaint under consideration. The last attack had now continued three months, without any relief from pain, though a great number of remedies had been used in vain. Each evacuation was followed by the excruciating pains already described, so that the poor man was often deprived of sleep for whole nights together, was reduced to despair, and almost entirely abandoned food, for fear of the sufferings attendant on the evacuation of the fæces.

In this state, he commenced the use of the "douche ascendante," by means of a syringe with a crooked pipe.* The first effect of this application was a diminution of the pains, and the reduction of the hæmorrhoidal tumours, so as to be reducible within the sphincter. The process was continued for three or four days, and the pains ceased entirely. He has now been five years free from complaint."

*Case* 2. "The second case was that of a man, 40 years of age, of plethoric constitution, who had been ten years affected with internal piles, without any discharge. A sedentary life, without any regular rest, for some months, had caused an hæmorrhoidal swelling, attended by inflammation, and, finally, by ulceration. To this state was added the excruciating pains in question. Having a water closet, of English construction, he contrived to have the jet of water thrown against the anus, which gave him instant relief, for a time. He renewed the application every time the pain came on, which was sometimes very often in the 24 hours, and always with the same effect. By persevering in this plan a month, or six weeks, the ulcerations, healed, the hæmorrhoids disappeared, and the pains ceased."

*Equitation.* Horse-exercise, in moderation, is recommended by Montegre, and justly, as one of the most powerful means of preventing and curing piles—though rough riding is a very common cause of their formation or exasperation. It is a valuable adjuvant to the cold water, in the painful nervous hæmorrhoids, of which we are now treating. It is evident, however, that great care is necessary, when commencing this kind of exercise, lest painful or inflamed tumours should be bruised or burst by the saddle, and thus increase the evil which the measure was designed to remedy. It is proper, at the same time, to state that Baron Larrey was long in the habit of causing soldiers affected with piles to ride on horseback, at full gallop, and he avers, that he never saw any bad effects follow, but generally, on the contrary, the most salutary consequences.

*Spasmodic Stricture of the Anus.* This severe complaint is very generally complicated with the nervous pain of hæmorrhoids, so often alluded to by Montegre. The spasmodic stricture itself causes pain—or which is more often the case, the pain causes spasmodic contraction of the sphincter ani, and thus renders the ejection of the fæces both difficult and dolorous. For this complaint, Montegre does not recommend the severe operation proposed and practised by M. Boyer. He avers that, of all the remedies, the "douche ascendante," or injections of

* The *douche ascendante* is merely the throwing of water forcibly *against the anus*, by means of a syringe or other instrument.—*Ed.*

cold water, are the most efficacious. For this purpose, the lavement should be thrown up when the inclination to stool takes place, which will render the ejection of the motion more easy, and prevent the painful torture which so commonly ensues, if this precaution be not taken. If this measure fail, it is time enough to have recourse to Boyer's operation of dividing the sphincter, as a last resource.

Where the stricture of the anus depends on the presence, and consequent irritation of permanent or organized hæmorrhoidal tumours, it is manifest that nothing but their removal will accomplish a cure.

*Treatment of Constipation.* This is a fruitful cause and frequent accompaniment of piles. Its removal is therefore an object of primary importance to the patient and practitioner. It is certain that every thing which increases the cutaneous transpiration, and the action of the absorbents scattered over the mucous membrane of the bowels, has a tendency to produce constipation. On this account, the hæmorrhoidarian should be cautioned against all violent exercises that cause much perspiration. The passive exercise of a carriage is well known as predisposing to confinement of the bowels—and even equitation is not quite free from the same objection, but it possesses many counterbalancing good qualities. A warm and soft bed is objectionable. Much depends on diet, in obviating constipation; but it is impossible to lay down any general rules in this respect, on account of the various and conflicting idiosyncrasies. It is certain, however, that, speaking generally, a rather stimulating food is proper in tendencies to constipation. But every person can judge of the kind of food that suits his constitution. Of laxative medicines, one of the best is sulphur, especially in the form of electuary with supertartrite of potash and honey or molasses. And when a purgative is required, we have found none answer so well as the common seidlitz powder taken early in the morning, and some warm tea an hour afterwards. It generally produces one loose, watery, and easy motion, with great relief to the hæmorrhoidal patient. But a still better remedy for constipation is obtained by keeping up a regular and due secretion from the liver and other glandular organs in the abdomen. For this purpose the infusion or decoction of taraxacum, with some tartrate of potash, or cream of tartar, is an excellent medicine, when continued a sufficient length of time. Minute doses of the pilula hydrargyri, with a quarter of a grain of ipecacuan, or an eighth of a grain of tartrite of antimony, at bed time, are generally necessary at the beginning.

*Lavements.* These are so habitually used by people afflicted with piles, especially on the continent, that it may seem treason to speak against them. It is not, however, against the principle of injections that we speak, but against the *mode* in which they are used. *Warm* lavements must invite blood to the parts, and thus increase rather than diminish the tendency to hæmorrhoids, although they give temporary solace, and prevent much irritation in the passing of the fæces. Injections of *cold* water, on the other hand, possess all the advantages, and none of the bad qualities of warm and relaxing lavements. They brace the mucous membrane of the gut, and repel the blood from the vessels of the part, thus very effectually checking the hæmorrhoidal enlargements which must sooner or later occur, when the hæmorrhoidal afflux is once fairly established. Montegre's opinion, on this point, is worthy of insertion here. "Les lavemens chauds ou tiédes produisent à la longue l'indolence et la paresse des intestins; quand on a depuis long temps l'habitude d'en user, on ne saurait aller à la garderobe sans en prendre, et souvent leurs secours doit etre repété deux, trois fois et plus, pour produire quelque effet." This is not the case, he remarks, with respect to cold injections. They give such tone to the bowels, that the mucous membrane secretes a sufficient quantity of fluid to facilitate the ejection of the fæcal remains. Our author relates a case in point, where a gentleman experienced an excruciating pain every time he had a motion, in consequence of a fissure in the gut. By the use of cold injections, whenever he felt a desire to have a stool, he completely recovered from this painful state, in the course of four months. Montegre advises that only half a syringe-ful should be thrown up, as it is not necessary or proper to fill more than the rectum. If the injection passes high up in the bowels it causes an evacuation of more than was destined to pass at that time, and thus gives rise to irritation about the anus.

*Constitutional Treatment.* Piles are among those diseases which it is sometimes dangerous to cure. Care should therefore be taken to distinguish those which ought, from those which ought not to be removed.

"Those hæmorrhoids," says Montegre, "which are of a constitutional nature, or which the constitution, as it were, requires, are generally of long standing—sometimes from youth—or they have replaced some grave and habitual affection—are hereditary—attended with well marked symptoms of plethora—take place from various and opposite exciting causes, or from no apparent cause at all—are preceded by general constitutional symptoms already described—are succeeded by a bettered condition of health, whether there be a discharge or not—and finally, are accompanied or followed by inconvenience when interrupted or suppressed—all these indicate a constitutional disorder which it is dangerous to meddle too rashly with."

The local or accidental hæmorrhoids are marked by symptoms the reverse of the above, and may be subjected to more active treatment. But it may be well to bear in mind that even these accidental hæmorrhoids, if they take place often, become at length associated with the animal economy, and, in fact, take on a constitutional nature.

Under certain circumstances, Montegre thinks, it may be advisable to provoke or bring on the hæmorrhoidal movement in the system; as, for instance, where there is a threatening of phthisis, of disease of the liver, or mental aberration. In illustration and confirmation of this precept, Montegre relates several cases from his own practice, and from the records of medicine. These, our limits will not permit us to dwell upon, in this place. It is proper to bear in mind, however, that this affection should not be provoked upon slight occasions —since, although in its simple form, it can hardly be called a disease, yet it may very frequently become complicated with a train of local evils which are amongst the most painful to which flesh is heir.

*Re-establishment of Suppressed Hæmorrhoids.* This may often be both proper and necessary, where the interruption or suppression is followed by symptoms which threaten the general health, or the function of some important organ. The means of re-establishing the hæmorrhoidal affection will readily suggest themselves to those who are acquainted with the general causes that commonly produce piles. These are, pediluvia and semicupia—leeches to the anus, with cupping-glasses in the neighbourhood of the rectum—purgatives, especially of aloes, rhubarb, colocynth, sulphate of soda—warm lavements, &c.

*Hygiene.* We must conclude this article, already rather extended, with a few general observations on the rules which should be given for the conduct of those who are subject to hæmorrhoids. It is not to be expected that any person will conform to all the rules which it may be proper for him to follow; yet still it is desirable that such rules should be known both to the patient and practitioner.

1. A temperate climate is that best adapted to the hæmorrhoidarian. A very hot and a very cold climate tend to produce constipation, and derange the digestive organs. Sudden vicissitudes of the atmosphere are unfavourable to a person disposed to hæmorrhoids, by deranging the cutaneous transpiration, and thus disturbing the functions of internal organs. Damp and low situations, for the same reason, are objectionable. The use of the tepid bath is salutary, but the hot bath is injurious, in Montegre's opinion. But of all preventive measures, cold water applied to the anus and neighbouring parts, after each motion, or oftener if necessary, is the most effectual security against piles. When hæmorrhoidal tumours protrude at stool, they should never be returned until they are carefully sponged with cold water. This practice should be pursued summer and winter without interruption. The clothing should be the same as for a person subject to rheumatism. It should be warm, to favour the insensible perspiration. Flannel is therefore proper. In respect to food, Montegre condemns the use of spices and stimulating matters, such as mustard, horse-radish, &c. In this point we do not quite agree with our able author; for we believe a moderate proportion of spices with our food is seldom injurious, and often assists the digestion. Spirituous liquors we condemn as much as Montegre. Heldebrandt, who has written well on the subject under discussion, observes that food and drink, taken hot, are injurious, and Montegre joins with him in opinion. We do not attach so much importance to this point as either of our authors. The grand object is to keep the digestive organs in a good state by the quantity and plainness of our food, by our temperance in drink, and by the regularity of our hours of waking, repose, and exercise. When medicine is necessary in addition to regimen, it should be such as corrects bad, and increases scanty secretions from the liver and other glandular organs of the abdomen, as well as from the cutaneous surface. The means of effecting this are well understood in this country.

We have now brought this article to a close—already too protracted, no doubt, in the opinion of the learned and experienced. But let it be remembered that we do not write entirely, nor even principally, for *these*. "Doctis indoctisque scribimus." It is the great object of this Journal to make that which is *true*, rather than that which is *new*, more generally known through all ranks of the profession than formerly. In this respect we are among the Levellers—not those who would level the profession to the ranks of the vulgar, or raise the vulgar to a level with the profession—but who would wish to see medical science itself more generally and more evenly distributed among the different denominations of its professors. If the Medico-chirurgical Review should prove contributory to this great and desirable end, its conductors will not have lived in vain.

